# Characteristics and outcomes of dengue infection in kidney transplant recipients: an integrative review

**DOI:** 10.1590/S1678-9946202668059

**Published:** 2026-09-28

**Authors:** Natasha de Lima Monteiro, Ana Carolina de Lima, Raquel Silveira Bello Stucchi, Marilda Mazzali, Marcos Vinicius de Sousa

**Affiliations:** 1 Universidade Estadual de Campinas, Faculdade de Ciências Médicas, Departamento de Clínica Médica, Área de Nefrologia, Laboratório de Investigação em Transplante, Unidade de Transplante Renal, Campinas, São Paulo, Brazil; 2 Universidade Estadual de Campinas, Faculdade de Ciências Médicas, Departamento de Clínica Médica, Área de Infectologia, Campinas, São Paulo, Brazil

**Keywords:** Dengue, Kidney transplantation, Virus disease, Acute kidney injury, Graft survival

## Abstract

Dengue is an arbovirus infection caused by the dengue virus, common in tropical and subtropical areas. It is mainly transmitted by bites from infected *Aedes aegypti* and *Ae. albopictus* mosquitoes, but donor-derived cases have been reported. In kidney transplant recipients with dengue, immunosuppressive therapy can affect their immune response and disease presentation. This study analyzed the characteristics and outcomes of kidney transplant recipients diagnosed with dengue. An integrative review was conducted, including case reports, series, case-control, and cohort studies. The PubMed/Medline search in August 2025 used this syntax: ((kidney transplantation) OR (renal transplantation)) AND (dengue). The articles were selected by two reviewers, with a third resolving discrepancies. The database search found 72 articles. After removing duplicates, 71 were screened. Overall, 41 articles were excluded because they did not address the study subject. In total, 30 articles underwent full-text review; eight were excluded-seven lacked kidney transplant recipients with dengue and one had insufficient case details on infected cases. This review included 22 studies from dengue-endemic countries regarding infection occurring from days to years after transplant. Bleeding was more common in infections occurring early post-transplant, possibly linking early infection to severe symptoms. The main symptoms were fever, myalgia, and headache, similar to those in immunocompetent hosts. Thrombocytopenia was frequent, likely due to viral infection and immunosuppressive drugs that affect bone marrow function. No studies assessed the effect of dengue on anti-human leukocyte antigen antibodies or on graft rejection. Graft loss was 4%, and most patients recovered from the disease.

## INTRODUCTION

Dengue is an infectious disease caused by the dengue virus (DENV), an arbovirus transmitted to humans via the bite of infected mosquitoes, mainly *Aedes aegypti* and *Ae. albopictus* ^1^ ^,^ ^2^. About half of the global population is at risk of dengue, with an estimated 100-400 million infections occurring each year, mainly in tropical and subtropical regions, particularly in the Caribbean, Central and South Asia, and South America^1^ ^,^ ^2^. DENV is a single-stranded RNA virus; its genome encodes seven non-structural proteins and three structural proteins, including the capsid, envelope, and membrane proteins. Structural proteins contribute to the architecture of the viral particles, whereas non-structural proteins are involved in the viral entry, replication, assembly, and pathogenesis^1^. The four DENV serotypes (DENV-1, DENV-2, DENV-3, and DENV-4) show varying distributions across countries. Most infected people have mild or no symptoms, but several host factors correlate with dengue severity. Pre-existing primed memory human leukocyte antigen (HLA) class I-restricted cross-reactive T cells have been linked to severity in secondary infections. In contrast, HLA class II, especially DRB1 alleles, seems protective. The balance between types 1 and 2 host immunity can also influence the clinical response to DENV, with type 1 linked to dengue fever and type 2 associated with severe disease^1^.

After an incubation of 4-10 days, infected hosts can show a wide range of symptoms depending on individual risk factors such as secondary infections, age, host immune status, and chronic conditions. The disease typically progresses in three phases: febrile, critical, and recovery. The febrile phase usually lasts two to seven days and is characterized by high-grade fever, rash, myalgia, arthralgia, and headache, with mild hemorrhagic manifestations. The critical phase lasts 24-48 h and is characterized by worsening leukopenia, a decline in platelet count, and increased capillary permeability. In this phase, warning signs can occur, including abdominal pain, persistent vomiting, clinical fluid accumulation, mucosal bleeding, lethargy, hepatomegaly, and an increase in hematocrit (reflecting hemoconcentration concurrent with a rapid decrease in platelet count). Severe dengue is defined as one or more of the following: plasma leakage leading to shock or fluid accumulation with respiratory distress, severe bleeding, or serious organ impairment. The recovery phase is characterized by the gradual reabsorption of extravascular fluid and normalization of blood cell counts. The World Health Organization recommends managing dengue cases by group^2^. Group A includes patients without warning signs who tolerate adequate oral fluid volumes and can be sent home. Group B includes patients with warning signs and comorbidities, such as renal failure, or those with comorbidities or social circumstances who require in-hospital observation or close monitoring. Group C includes patients who require emergency treatment and immediate advanced care because of severe dengue. 

In kidney transplant recipients with dengue, immunosuppressive therapy may affect their immune response to the infection, especially when immunosuppression is excessive. Additionally, medications such as antiproliferative drugs and steroids can modify the clinical presentation and laboratory findings of the disease^3^. Previous studies have shown a higher incidence of symptomatic dengue in transplant recipients than that in the general population but lower rates of severe dengue in immunosuppressed hosts^4^. Viral infections have been linked to kidney transplant complications and graft rejection, but the impact of arboviral infections on kidney transplant recipients’ outcomes remains under study. This research aims to evaluate previous studies to assess the baseline characteristics of kidney transplant recipients diagnosed with dengue, as well as aspects related to the disease, including clinical and laboratory manifestations, immunosuppressive management, and outcomes. It also reports cases of acute rejection following dengue infection, as well as the treatments instituted for these cases.

## MATERIALS AND METHODS

This integrative review was conducted according to the PRISMA guidelines^5^. The eligibility criteria were based on PICOS framework^6^. This study focused on kidney transplant recipients diagnosed with dengue. No interventions or comparisons were performed. The assessed outcomes included post-transplant timing for infection, signs and symptoms, dengue clinical staging, laboratory results, infected recipients’ demographic profile, baseline immunosuppressive therapy and management during infection, and the following disease outcomes: acute kidney injury (AKI) and graft loss. AKI is defined as an abrupt decrease in kidney function occurring within seven days, detected by increased serum creatinine or reduced diuresis due to various etiologies^7^. Case reports, series, case-control, and cohort studies were included in this review. The search strategy involved querying PubMed/Medline in August 2025 using the syntax ((kidney transplantation) OR (renal transplantation)) AND (dengue). Results were imported into Rayyan™, a web app for systematic reviews^8^. After removing duplicates, articles were screened by title and abstract, and final inclusion was determined by full-text review.

Titles and abstracts were screened by two independent reviewers to determine eligibility for full-text review. A third author, M.V.S., resolved any discrepancies. Selection was independently carried out by three researchers. Duplicates and articles that did not evaluate kidney transplant recipients with dengue were excluded. The available data-including study period, research location, number of recipients, time post-transplant at dengue diagnosis, signs and symptoms, clinical dengue stage, laboratory findings, recipient sex, number of deceased donors, immunosuppressive therapy at baseline and during infection management, and outcomes such as AKI, graft loss, and recovery-were independently extracted by the reviewers. The data were organized on an Excel™ sheet for analysis.

## RESULTS

The database search retrieved 72 articles, including two duplicates. This study included one additional record in other sources. After excluding the duplicates, this review screened 71 publications, excluding 41 articles for failing to address the study subject. This study found 30 articles to be eligible for full-text review; excluding eight: seven included no kidney transplant recipients with dengue and one lacked details on infected cases. This review included 22 studies (Figure 1). 

**Figure 1:**
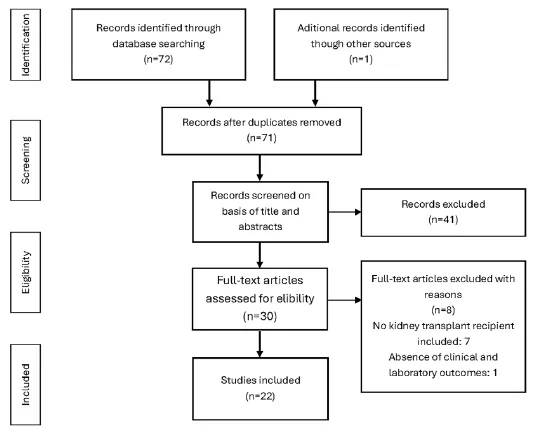
PRIMA flow diagram showing the identification, screening, eligibility, and inclusion of the included studies.

All included articles had undergone peer review, being published in Portuguese or English from 2004 to 2021. Most studies were carried out in India (n=7, 31.8%), followed by Brazil (n=5, 22.7%) and Singapore (n=5, 22.7%). In total, seven studies (31.8%) were single-case reports, and the largest study included 102 infected kidney transplant recipients^9^. The total number of recipients across the included studies equaled 342, most of whom were male (n=215, 63%). The chosen studies included no recipients of multi-organ transplants. Only one study reported a case of donor-derived dengue infection transmitted by the same donor to two kidney transplant recipients^10^. At the time of dengue diagnosis, most recipients were on immunosuppression with a calcineurin inhibitor (n=292, 85%), an antimetabolite (n=272, 84%), and steroids (n=306, 99%). A small number of patients used mammalian target of rapamycin inhibitors at the time of infection (n=25, 8%). Only 12 studies (54%) reported immunosuppressive management during infection, involving 101 patients, of whom 17 (16%) maintained their immunosuppressive therapy unchanged, 41 (41%) had their therapy adjusted, including dose reductions or changes in immunosuppressive agents, and 43 (43%) had their therapy partially or completely withdrawn. The class of immunosuppressive drugs most frequently adjusted during acute infection was antimetabolites, mainly mycophenolate (Table 1).

**Table 1:** Transplant-related data from included studies regarding recipients with dengue infection after kidney transplantation.

Article	Country Year	Total	Male, n (%)	DD, n (%)	CNI, n (%)	AM, n (%)	MTORi, n (%)	Steroids, n (%)	IMS management, n (%)	AKI, n (%)	Graft loss, n (%)
Bansal *et al.* ^14^	India 2021	4	4 (100)	0 (0)	TAC: 4 (100)	MPA: 4 (100)	0 (0)	4 (100)	MPA withdrawn: 4 (100)	2 (50)	0 (0)
Tan *et al.* ^20^	Singapore 2021	31	15 (48)	18 (58)	TAC: 14 (45) CsA: 12 (39)	MPA: 18 (58) AZA: 6 (19)	4 (13)	31 (100)	Maintenance: 8 (26) / CNI adjustment: 9 (34) AM reduction: 5 (20) / AM withdrawn: 11 (46)	19 (61)	na
Sim *et al.* ^10^	Singapore 2020	2	2 (100)	1 (100)	TAC: 2 (100)	MPA: 2 (100)	0 (0)	2 (100)	na	na	na
Meshram *et al.* ^21^	India 2020	31	22 (70)	10 (32)	TAC: 29 (93) CsA: 1 (3)	MPA: 25 (80) AZA: 4 (13)	0 (0)	31 (100)	na	20 (64)	2 (6)
Ng *et al.* ^16^	Singapore 2019	1	0 (0)	0 (0)	TAC: 1 (100)	MPA: 1 (100)	0 (0)	1 (100)	MPA withdrawn: 1 (100)	1 (100)	0 (0)
Thomas *et al.* ^19^	India 2019	12	11 (92)	na	TAC: 10 (83) CsA: 1 (8)	MPA: 10 (83) AZA: 1 (8)	0 (0)	na	na	11 (92)	na
Fernandes-Charpiot *et al.* ^22^	Brazil 2019	39	17 (44)	22 (56)	27 (70)	MPA: 22 (56)	15 (39)	39 (100)	Adjustment: 12 (31)	23 (59)	1 (3)
Ribeiro *et al.* ^23^	Brazil 2019	19	5 (26)	11 (58)	TAC: 19 (100)	AZA: 3 (16) MPA: 7 (37)	4 (21)	19 (100)	Maintenance: 8 (42) / TAC reduction: 1 (5) MPA reduction: 7 (100) / AZA reduction: 3 (100), MTORi reduction: 1 (25)	11 (58)	0 (0)
Pinsai *et al.* ^24^	Thailand 2019	13	7 (54)	6 (42)	TAC: 8 (61) CsA: 5 (38)	MPA: 13 (100)	0 (0)	13 (100)	TAC switched to CsA: 1 (13) / MPA switched to AZA: 1 (8) MPA withdrawn: 12 (92)	10 (77)	1 (8)
Weerakody *et al.* ^25^	Sri Lankan 2018	1	0 (0)	0 (0)	0 (0)	AZA: 1 (100)	0 (0)	1 (100)	AZA withdrawn: 1 (100)	1 (100)	0 (0)
Subbiah *et al.* ^27^	India 2018	1	1 (100)	0 (0)	TAC: 1 (100)	MPA: 1 (100)	0 (0)	1 (100)	MPA withdrawn: 1 (100)	1 (100)	0 (0)
Subbiah *et al.* ^26^	India 2018	20	20 (100)	0 (0)	TAC: 19 (95) CsA: 1 (5)	na	na	na	Total withdrawn: 12 (60)	8 (40)	0 (0)
Fernandes *et al.* ^28^	Brazil 2017	11	4 (36)	9 (82)	TAC: 7 (64) CsA: 3 (27)	MPA: 7 (64) AZA: 3 (27)	1 (9)	9 (82)	na	6 (54)	na
Costa *et al.* ^29^	Brazil 2015	10	5 (50)	3 (30)	TAC: 5 (50) CsA: 5 (50)	MPA: 9 (90)	1 (10)	9 (90)	na	3 (30)	2 (20)
Nasim *et al.* ^9^	Pakistan 2013	102	75 (74)	0 (0.0)	TAC: 21 (21) CsA: 58 (57)	MPA:27 (26) AZA: 67 (66)	0 (0)	102 (100)	na	68 (67)	na
Prasad *et al.* ^30^	India 2012	8	4 (50)	na	TAC: 3 (38) CsA: 5 (62)	MPA: 8 (100)	0 (0)	8 (100)	na	4 (50)	0 (0)
Tangnararatchakit *et al.* ^17^	Thailand 2012	1	0 (0)	0 (0)	CsA: 1 (100)	MPA: 1 (100)	0 (0)	1 (100)	MPA reduction: 1 (100)	0 (0)	0 (0)
Park *et al.* ^18^	Korea 2008	1	0 (0)	0 (0)	TAC: 1 (100)	0 (0)	0 (0)	0 (0)	Maintenance: 1 (100)	1 (100)	0 (0)
Azevedo *et al.* ^31^	Brazil 2007	27	18 (67)	6 (22)	TAC: 4 (15) CsA: 17 (63)	MPA: 14 (63) AZA: 13(48)	0 (0)	27 (100)	na	na	na
Renaud *et al.* ^33^	Singapore 2007	6	4 (67)	na	TAC: 2 (33) CsA: 4 (67)	MPA: 3 (50)	0 (0)	6 (100)	na	0 (0)	0 (0)
Tan *et al.* ^13^	Singapore 2005	1	1 (100)	0 (0)	TAC: 1 (100)	MPA: 1 (100)	0 (0)	1 (100)	MPA withdrawn: 1 (100)	0 (0)	0 (0)
Chacko *et al.* ^32^	India 2004	1	0 (0)	na	CsA: 1 (100)	AZA: 1 (100)	0 (0)	1 (100)	na	1 (1)	0 (0)
Total		342	215 (63)	86 (27)	292 (85)	272 (84)	25 (8)	306 (99)	Maintenance: 17 (16) / Adjustment: 41 (41) Partial or total withdrawn: 43 (43)	190 (61)	6 (4)

N = number; na = not available; DD = deceased donor; CNI = calcineurin inhibitor; AM = antimetabolit; MTORi = mammalian target of rapamycin inhibitor; IMS = immunosuppression; AKI = acute kidney injury; TAC = tacrolimus; CsA = cyclosporine; MPA = mycophenolic acid; AZA = azathioprine.

Among the included studies, 20 (91%) analyzed the incidence of AKI due to dengue infection, reporting a 61% incidence. However, most recipients recovered graft function, and only 6 (4%) showed graft loss due to dengue infection (Table 1). Results of anti-HLA antibody screening or episodes of rejection diagnosed after dengue infection were not reported by the included studies.

The post-transplant period for infection varied across studies, ranging from one day to 17 years (Table 1). Fever was the most common symptom (n=266, 83%), followed by myalgia (n=154, 48%) and headache (n=66, 20%). Although most reports observed thrombocytopenia (n=224, 86%), hemorrhagic manifestations were rare, appearing in only two studies. White blood cell reduction also occurred frequently (n=142, 55%), whereas anemia occurred less often (n=11, 4%). No study classified dengue by clinical stage. However, of the 91 classified cases, 25 (28%) recipients presented with stage B; 22 (24%), with stage C; and 44 (48%), with stage D. Most patients recovered from dengue infection (n=325, 95%), whereas death occurred in 17 cases (5%) (Table 2).

**Table 2:** Dengue-related clinical characteristics and outcomes.

Article	Total	Time post-transplant	Clinical staging group, n (%)	Signs and symptoms, n (%)	Blood cell reduction, n (%)	Recovery, n (%)	Death, n (%)
Bansal *et al.* ^14^	4	6 (2-7) d	na	Fever: 4 (100.0)	WBC: 2 (50) / PLT: 4 (100)	4 (100)	0 (0)
Tan *et al.* ^20^	31	na	D: 3 (10)	Fever: 27 (87) / Myalgia: 8 (26) / Headache: 7 (23)	na	30 (97)	1 (3)
Sim *et al.* ^10^	2	5 d	na	na	PLT: 1 (50)	2 (100)	0 (0)
Meshram *et al.* ^21^	31	1.5 (1-7) y	B: 20 (64) / C: 9 (29) / D: 2 (7)	Fever: 29 (93) / Chills: 21 (68) / Headache: 12 (39)	RBC: 3 (9) / WBC: 18 (58) / PLT: 29 (94)	31 (100)	0 (0)
Ng *et al.* ^16^	1	11 w	na	Joint pain / Myalgia / Diarrhea	WBC / PLT	yes	no
Thomas *et al.* ^19^	12	89 m (9 d-328 m)	na	na	na	11 (92)	1 (8)
Fernandes-Charpiot *et al.* ^22^	39	65 ± 62 m	D: 6 (15)	Fever: 24 (61) / Myalgia: 32 (82) / Malaise: 27 (69)	WBC: 26 (67) / PLT: 29 (74)	37 (95)	2 (5)
Ribeiro *et al.* ^23^	19	72 ± 57 m	B: 4 (21) / C: 13 (68) / D: 2 (10)	Myalgia: 12 (63) / Headache: 9 (47) / Fever: 8 (42)	RBC: 2 (10) / PLT: 15 (79)	19 (100)	0 (0)
Pinsai *et al.* ^24^	13	24 (6-122) m	D: 2 (15)	Fever: 13 (100) / Myalgia: 9 (69) / Headache: 5 (38)	na	13 (100)	0 (0)
Weerakody *et al.* ^25^	1	17 y	D	Fever / Myalgia / Headache	RBC / WBC / PLT	yes	no
Subbiah *et al.* ^27^	1	na	D	Fever / Myalgia / Fatigue	WBC / PLT	yes	no
Subbiah *et al.* ^26^	20	378.5 (1-3249) d	D: 2 (10)	Fever: 19 (95) / Myalgia: 13 (65) / Headache: 6 (30)	WBC: 10 (50) / PLT: 18 (90)	19 (95)	1 (5)
Fernandes *et al.* ^28^	11	3.6 y (1 m-9 y)	D: 2 (18)	Fever: 11 (100) / Myalgia: 10 (91) / Headache: 6 (54)	WBC: 4 (37) / PLT: 9 (82)	11 (100)	0 (0)
Costa *et al.* ^29^	10	44.5 m (5 d-166 m)	D: 4 (40)	Myalgia: 10 (100) / Headache: 10 (100) / Fever: 9 (90)	RBC: 3 (30) / PLT: 7 (70)	10 (100)	0 (0)
Nasim *et al.* ^9^	102	1.3 y (2 d-14 y)	D: 12 (12)	Fever: 82 (80) / Myalgia: 23 (22) / Gastrointestinal symptoms: 35 (34)	WBC: 72 (71) / PLT: 97 (95)	95 (93)	7 (7)
Prasad *et al.* ^30^	8	22 (1-120) m	D: 3 (37)	Fever: 8 (100) / Myalgia: 8 (100) / Headache: 8 (100)	PLT: 8 (100)	5 (63)	3 (37)
Tangnararatchakit *et al.* ^17^	1	13 d	D	Diarrhea / Ascitis / Pleural effusion	PLT	yes	no
Park *et al.* ^18^	1	156 m	B	Headache / Fever / Myalgia / Diarrhea	WBC / PLT	yes	no
Azevedo *et al.* ^31^	27	64 ± 61 m	D: 1 (4)	Fever: 27 (100) / Myalgia: 24 (89) / Malaise: 20 (74)	na	26 (96)	1 (4)
Renaud *et al.* ^33^	6	53 ± 37 m	na	na	WBC: 5 (83)	6 (100)	0 (0)
Tan *et al.* ^13^	1	5 d	D	Fever / Bleeding / Abdominal pain	RBC / WBC / PLT	yes	no
Chacko *et al.* ^32^	1	18 m	D	Myalgia / Headache / Fever	RBC / PLT	no	yes
Total	342	1d-17 y	B: 25 (28) / C: 22 (24) / D: 44 (48)	Fever: 266 (83) / Myalgia: 154 (48) / Headache: 66 (20)	RBC: 11 (4) / WBC: 142 (55) / PLT: 224 (86)	325 (95)	17 (5)

N = number; na = not available; d = day; w = week; m = month; y = year; RBC = red blood cell; WBC = white blood cell; PLT = platelet.

## DISCUSSION

This revision included studies from dengue-endemic countries such as India, Brazil, Singapore, Thailand, Sri Lanka, Pakistan, and South Korea. Dengue remains hyperendemic in tropical and subtropical regions globally, mainly in urban and semi-urban areas. The global incidence of dengue is estimated at 100-400 million cases annually, with a rapid increase in recent years^11^. According to the Global Observatory on Donation and Transplantation, 91 countries (some of which in dengue-endemic areas) reported 173,727 solid organ transplants in 2024^12^. Therefore, kidney transplant recipients in areas endemic for dengue are exposed to an increased risk of developing the disease. Knowledge of the potential effects of dengue in this population is essential for developing strategies to prevent, manage the acute phase of the disease, and treat its main complications. Although the search strategies in this review did not specify a publication period, all included studies were published in the last 20 years. This fact highlights the recently growing relevance and interest in this topic, along with a better understanding of dengue behavior among kidney transplant recipients. 

The time elapsed since transplantation at the time of infection was highly variable, ranging from days to several years, indicating that dengue can impact any stage after transplantation. Almost half of the included studies reported a dengue case diagnosed within the first month after transplant. Tan *et al*.^13^ described a case of symptomatic donor-derived dengue in a recipient within the first week after transplant (presenting with bleeding symptoms), which showed complete resolution of the infection along with preserved graft function. Sim et al.^10^ also reported an early dengue infection following a transplant, linked to the donor who had a negative DENV viremia but positive DENV viruria, transmitting the virus to two kidney transplant recipients. Bansal *et al*.^14^ reported four cases of kidney transplant recipients diagnosed with dengue shortly after their transplants, including one with a confirmed infection in the donor. Among these patients, two developed graft dysfunction and thrombocytopenia requiring platelet transfusions, but all cases resolved completely. In this review, bleeding was more common during early post-transplant infections. This highlights a possible link between early infection and increased severity of the clinical symptoms of the disease. This finding resembles that of previous studies^15^.

The main symptoms in infected transplant recipients were fever, myalgia, and headache. These findings resemble those seen in immunocompetent hosts. Gastrointestinal symptoms, such as diarrhea, were also frequent^9^ ^,^ ^13^ ^,^ ^16^ ^-^ ^18^. The most common laboratory findings were thrombocytopenia and a reduction in white blood cell count, similar to those seen in non-transplant patients. However, Thomas*et al*.^19^ showed the lowest white blood cell nadir in transplant recipients than in non-transplant chronic kidney disease patients and participants with normal renal function. Thrombocytopenia is usually more severe in kidney transplant recipients because of the impact of immunosuppressive drugs on bone marrow suppression^19^. The main immunosuppression management in the included studies was the reduction or withdrawal of antimetabolites. Most of the included studies reported cases in clinical staging group D, which accounted for nearly half of patients with documented disease stages^9^ ^,^ ^13^ ^,^ ^17^ ^,^ ^20^ ^-^ ^32^. Despite the high prevalence of severe dengue among kidney transplant recipients, most reported cases in this review resulted in clinical recovery, similar to the benign course described in previous studies^33^.

Dengue infection is associated with several renal manifestations, such as serum electrolyte abnormalities, proteinuria, changes in urinary sediment, and acute kidney injury^34^. The incidence of acute kidney injury in patients with dengue is variable, and some risk factors can be identified, such as older age, male sex, previous stroke or chronic kidney disease, gastrointestinal bleeding, concurrent bacteremia, low hemoglobin and serum albumin levels, prolonged prothrombin time, and elevated liver enzymes^34^. Kidney grafts seem to be particularly vulnerable to injury during dengue fever since they are susceptible to nephrotoxic drug effects, ischemia-reperfusion injury, and episodes of acute rejection^35^. In addition to these effects, the cytokine profile induced by viral infection and the immune response triggered by the virus may increase the risk of kidney graft rejection, especially when immunosuppressive therapy must be reduced. These factors may contribute to chronic kidney transplant rejection, a silent and progressive immune response that causes ongoing graft damage and function decline, affecting long-term graft survival^36^. While this study showed a higher rate of graft function recovery and a lower rate of graft failure among infected recipients, explaining the potential long-term effects of dengue infection on renal graft survival requires further research.

## CONCLUSIONS

Dengue infection in kidney transplant recipients shows some similarities to cases in immunocompetent hosts. However, it tends to be linked with more severe symptoms in the early post-transplant phase. Laboratory abnormalities are generally more pronounced in transplant recipients because of the impact of immunosuppressive therapy on bone marrow function. These grafts are particularly vulnerable to injury during dengue, but the influence of the disease on anti-HLA antibodies and graft rejection remains unclear. In this review, graft failure was uncommon, and most patients fully recovered from the infection.

## Data Availability

The complete anonymized dataset supporting the findings of this study is included within the article itself.
